# Long-Term Outcomes of Cementless Versus Hybrid Cemented Total Knee Arthroplasty: A Minimum 10-Year Follow-Up

**DOI:** 10.3390/jcm14093134

**Published:** 2025-04-30

**Authors:** Lukas Rabitsch, Klemens Vertesich, Alexander Giurea, Reinhard Windhager, Richard Lass

**Affiliations:** Department of Orthopaedics and Trauma Surgery, Medical University of Vienna, Spitalgasse 23, 1090 Vienna, Austria; lukas.rabitsch@meduniwien.ac.at (L.R.); klemens.vertesich@meduniwien.ac.at (K.V.); alexander.giurea@meduniwien.ac.at (A.G.); richard.lass@meduniwien.ac.at (R.L.)

**Keywords:** arthroplasty, total knee arthroplasty, cementless fixation, cemented fixation, complications, aseptic loosening

## Abstract

**Background**: Although cemented total knee arthroplasty (TKA) is considered the standard fixation technique, the emerging trend toward cementless fixation has created the need for a detailed comparison. In a previous study, we reported the 5-year results comparing cementless and hybrid cemented TKAs using the same implant design. The purpose of this study was to assess the long-term follow-up at a minimum of 10 years. **Methods**: A retrospective analysis was performed on 120 TKAs (60 cementless, 60 hybrid cemented) conducted between 2003 and 2007 using the e.motion posterior cruciate-retaining knee prosthesis with a floating-platform mobile polyethylene bearing (Aesculap, Tuttlingen, Germany). Demographic and clinical data were collected; radiographic follow-up was performed with attention to signs of loosening, while complications and revision surgery were assessed using competing risk analysis. Operative time was recorded as an indicator of surgical efficiency. **Results**: At 10 years, 59 TKAs (54 patients) were available for long-term follow-up. Both fixation groups demonstrated significant improvement in Knee Society Scores (KSSs) compared to preoperative values (*p* < 0.001). However, there was no significant difference in KSSs between the two groups at 10 years follow-up (*p* = 0.480). The 10-year cumulative incidence of revision was 8.4% in both groups (*p* = 0.721), and that of aseptic loosening was identical at 3.4% (*p* = 0.967). Although radiolucent lines were noted in three tibial components of the cementless group, the difference was not statistically significant (*p* = 0.075). Notably, the cementless group demonstrated a significantly shorter operative time with a mean difference of 10 min (*p* = 0.017). **Conclusions**: At a minimum follow-up of 10 years, there were no significant differences between the hybrid cemented and cementless groups in revision rates, cumulative incidences, clinical scores, or radiological signs of loosening, confirming the long-term effectiveness of both fixation methods in clinical practice.

## 1. Introduction

Despite the excellent survival rates and clinical outcomes of TKA, there is an ongoing debate regarding the optimal fixation of components. As the number of TKAs performed annually increases and the patient population trends younger, the longevity of the prostheses without the need for revision becomes an increasingly important consideration [1,2,3]. This concern is particularly relevant, as the most common indication for revision surgery is aseptic loosening, which occurs at higher rates in younger patients [4].

While cemented fixation remains the gold standard—particularly in older patients with low bone density—cementless TKAs have been developed as an alternative for younger, more active individuals, offering advantages such as bone stock preservation, avoidance of cement debris, shorter operative times, and potential for long-term biological fixation, which may enhance implant survival rates [5]. However, in the early stages of development, cementless TKA implants were associated with higher rates of implant failure due to poor implant design [6,7]. Design advancements, including bioactive materials and osteoconductive coatings, have overcome the early limitations of cementless implants, resulting in better survival rates and lower revision rates due to improved bone ingrowth [8]. For instance, Nam et al. [9] analyzed data from the American Joint Replacement Registry and found that 3-dimensional (3D) printed cementless tibial baseplates had superior survivorship and lower revision rates compared to both aggregate cementless and cemented tibial baseplates after 5 years. In a more recent study, Hannon et al. [10] reported in a prospective randomized controlled trial (RCT) a 6-year survivorship of 100% for both cementless and cemented TKAs of the same design. In addition, Van der Lelij et al. [11] conducted an RCT on cementless 3D-printed TKAs using radiostereometric analysis. The study showed no significant difference in mean migration at the five-year follow-up compared to the cemented implants.

Although cementless TKA implants have shown promising short-term performance and positive clinical outcomes, the current literature lacks comprehensive mid- to long-term follow-up data. Therefore, this study aims to address this gap. Previously, we reported the minimum 5-year results from this study population comparing cementless and hybrid cemented TKAs of the same design. At a mean follow-up of 6 years, there were no significant differences in revisions, clinical scores, or radiological signs of loosening between cemented TKAs and hybrid cemented TKAs [12].

The main aim of this study was to provide long-term follow-up data from the previous case-controlled study and compare the implant survivorship, clinical outcomes, and radiographic findings of cementless and hybrid cemented TKA implants with identical designs. The main hypothesis was that cementless TKAs would have similar long-term outcomes compared to hybrid cemented TKAs at a minimum follow-up of 10 years postoperatively.

## 2. Materials and Methods

Following approval from the ethical committees of the Medical University of Vienna and the General Hospital of Vienna, we retrospectively analyzed a consecutive series of 120 TKAs performed between July 2003 and January 2007 at the Department of Orthopaedics, Medical University of Vienna. As previously detailed in a case–control study [12], the cohort was divided equally into two groups: 60 cementless TKAs and 60 hybrid cemented TKAs. The selection of the fixation method was based on the operating surgeon’s established practice, thereby reflecting routine clinical decision-making. Inclusion criteria encompassed patients diagnosed with idiopathic arthritis, post-traumatic arthritis, or rheumatoid arthritis. Exclusion criteria were a history of revision surgery, prior joint infection, any primary or secondary cancer within the past 5 years, psychosocial disorders limiting rehabilitation, a valgus or varus deformity greater than 20°, or a flexion deficit exceeding 20°. The primary diagnosis was idiopathic arthritis (*n* = 106; 88.3%) followed by post-traumatic arthritis (*n* = 6; 5%), rheumatoid arthritis (*n* = 4; 3.3%), previous unicompartmental knee arthroplasty (*n* = 3; 2.5%), and avascular necrosis (*n* = 1; 0.8%). The mean age at surgery was 66.93 years (range: 33–90), with a total of 81 female and 39 male patients. Notably, the hybrid cemented group had a significantly higher proportion of female patients compared to the cementless group (76.67% vs. 58.33%, *p* = 0.032). The mean body mass index (BMI) was 30.28 kg/m^2^, ranging from 19 to 51. The mean operative time was significantly shorter in the cementless group (125.7 min; range: 80–175) compared to the hybrid cemented group (135.7 min; range: 95–200) (*p* = 0.017). Apart from the differences in operative time and gender distribution, no other significant demographic or clinical differences were observed between the two cohorts (Table 1).

### 2.1. Surgical Procedure

All operations were performed by a team of experienced orthopedic surgeons in our department, following a standardized protocol to ensure consistency. A standard medial parapatellar approach was utilized in every case, with the OrthoPilot navigation system ensuring neutral mechanical alignment; no kinematic alignment techniques were employed, and alignment goals remained identical across groups. Following prophylactic antibiotic administration, a tourniquet was applied for a maximum of 120 min to optimize surgical conditions. All 120 TKAs were performed with the e.motion posterior cruciate-retaining knee prosthesis with a floating-platform mobile polyethylene bearing (Aesculap, Tuttlingen, Germany). Sixty TKAs were performed uncemented using the e.motion prosthesis with a 350 μm titanium plasma-sprayed coating and an additional dicalcium phosphate dihydrate (μ-CaP) surface layer. The remaining 60 TKAs underwent a hybrid fixation technique, which involved a cemented tibial component and a cementless femoral component. In all cases, the patella was resurfaced with a cemented polyethylene component. Palacos R cement (Heraeus Medical GmbH, Wehrheim, Germany) was used for all patients. The implant design was the same for all patients, except for the surface of the uncemented tibial tray, as previously mentioned. Postoperative care included early mobilization on crutches and partial weight-bearing with approximately 50% of body weight on the operated leg for a period of 6 weeks [12].

### 2.2. Study Parameters

Preoperative characterization of the study population included collecting patient-specific parameters, including primary diagnosis, age, gender, weight, height, BMI, date of surgery, and the operated side (Table 1). Postoperative clinical and radiographic assessment followed the department’s standardized postoperative assessment protocol, which includes assessments at 6, 12, and 24 weeks, 1 year, 2 years, and every 5 years thereafter. Patients who did not attend follow-up were contacted by telephone to gather patient-reported outcomes. Deceased patients were identified using the hospital’s internal information software and a death registry inquiry. For clinical and functional evaluation, the KSS was measured at each follow-up. Revision surgery data, such as the timing and reason for revision, were extracted from surgical reports, discharge summaries, or follow-up examinations. Radiographs taken at each follow-up were analyzed for radiolucent lines or any alterations in the position of the implant. Radiolucent lines were evaluated based on their location, width (measured in millimeters), and progression, according to the guidelines established by the Knee Society [13,14]. The system assigns numerical scores for the prosthetic interface in different zones. The total score for each component was then calculated by adding up the widths of the radiolucent lines in all designated zones and rated as follows: Scores of 4 or less were considered non-progressive and typically insignificant. Scores between 5 and 9 indicated the need for careful monitoring for any increase. Scores of 10 or above indicated a potential or imminent failure of the component. All analyses were performed by a non-surgeon investigator.

### 2.3. Data Analyses

Descriptive data analysis was performed by calculating means, standard deviations (SDs), medians, ranges, and ratios based on the distribution of the measurements. In addition, the KSSs and results from the “Knee Society endorsed roentgenographic knee evaluation system” were systematically analyzed at each follow-up visit. Age and BMI at baseline and pre- and post-operative scores were compared using two-tailed independent *t*-tests. The Kolmogorov–Smirnov test confirmed a normal distribution. Pearson’s Chi-Square analysis was used for categorical data, such as gender or operative side.

Given the high rate of death in the study cohort, a competing risk analysis was conducted, treating death as a competing event. Accordingly, the cumulative incidences of revisions were estimated using the entire cohort, acknowledging the decreasing number at risk over time due to patient attrition. For instance, although clinical outcomes at 10 years were available for 59 TKAs, the revision risk estimates reflect time-to-event analysis in the full cohort. To evaluate differences between the two groups, Gray’s test was used [15]. All statistical tests were two-tailed, and a *p*-value of ≤0.05 was considered significant. All statistical calculations were performed using IBM SPSS Statistics version 29.0 and XLSTAT version 2023.3.1.

## 3. Results

### 3.1. Evaluable Cases and Demographic Comparison of Final Cohorts

**Figure 1:**
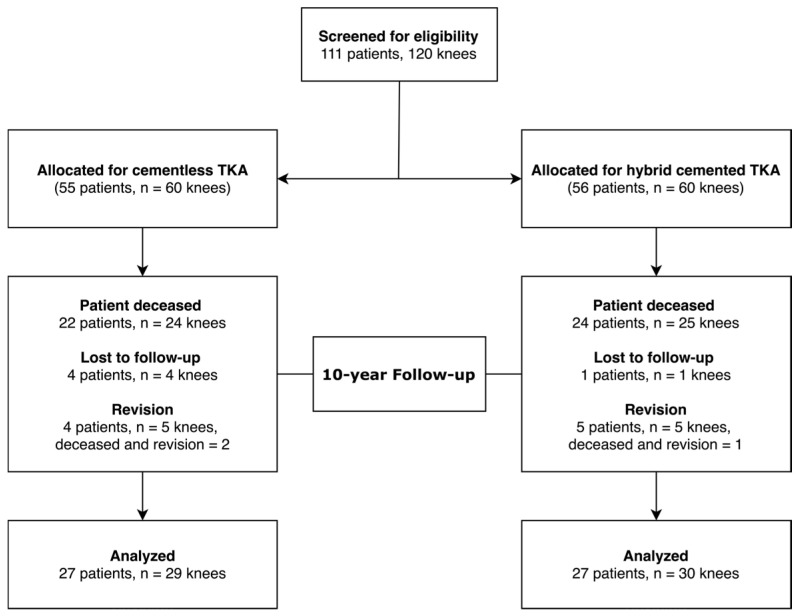
Flow diagram of patients in the study.

From the initial cohort of 120 TKAs (111 patients), 46 patients (49 TKAs, 40.8%) died from causes unrelated to the index procedure before the 10-year follow-up. An additional five patients (5 TKAs, 4.16%) were unable to be reviewed and therefore classified as “lost to follow-up”. Notably, no complications or revisions were reported at their last assessment. Consequently, complete follow-up data were available for 54 patients, accounting for 59 TKAs. Among these, 30 TKAs (50.8%) were hybrid cemented and 29 (49.2%) were cementless, with a mean follow-up period of 15.34 years (range, 10 to 20) (Figure 1).

To assess potential attrition bias and ensure group comparability, a demographic comparison was performed between the evaluable cementless (*n* = 29) and hybrid cemented (*n* = 30) TKAs. The mean age at surgery was 61.8 years (range: 49–77.5) in the cementless group and 63.8 years (range: 35–78) in the hybrid cemented group (*p* = 0.234). The gender distribution was similar, with 23 (79.41%) female patients in the cementless group versus 23 (76.7%) in the hybrid cemented group (*p* = 0.807). Mean BMI was 30 kg/m^2^ (range: 19–37) in the cementless group and 30.5 kg/m^2^ (range: 23–46) in the hybrid cemented group (*p* = 0.915). In addition, the distribution of operated knees (left/right), operative time, and the distribution of primary diagnoses did not differ significantly between the groups (*p* = 0.732, *p* = 0.447, and *p* = 0.353, respectively). These findings indicate that, despite the attrition observed over the 10-year follow-up, the two cohorts remained demographically comparable (Table 2).

### 3.2. Revisions

In the entire cohort, a total of 20 complications required revision surgery, including 11 revisions in the hybrid cemented group and 9 in the cementless group. Polyethylene failure was the predominant cause of revision, with 4 cases in the hybrid cemented group and 3 in the cementless group, presenting median revision times of 186 months (IQR: 167–190) and 152 months (IQR: 151–161), respectively. Aseptic loosening was the second most common cause, resulting in 3 revisions in each group, with a median time to revision of 25 months (IQR: 14–57) for the hybrid cemented group and 10.5 months (IQR: 3–60) for the cementless group. In the hybrid cemented group, one case involved combined tibial and femoral component loosening at 122 months, whereas the other two cases were isolated tibial failures at 14 and 36 months, respectively. In the cementless group, one tibial component loosening occurred late at 188 months, another early revision at 3 months was attributed to a confirmed postoperative nickel allergy, and a third patient with a high BMI (35 kg/m^2^) required revision at 18 months postoperatively. Limited mobility accounted for 4 revisions: 1 in the hybrid cemented group and 3 in the cementless group, with a median time to revision of 23 months (IQR: 23–23) for the hybrid cemented group and 5.5 months (IQR: 4–10) for the cementless group. Additionally, infection led to 3 revisions in the hybrid cemented group, with a median time to revision of 46 months (IQR: 11–94). Gray’s test indicated that there was no statistically significant difference in the revision rates between the two groups for any endpoint (Table 3).

### 3.3. Cumulative Incidence and Competing Risk Analysis

**Figure 2:**
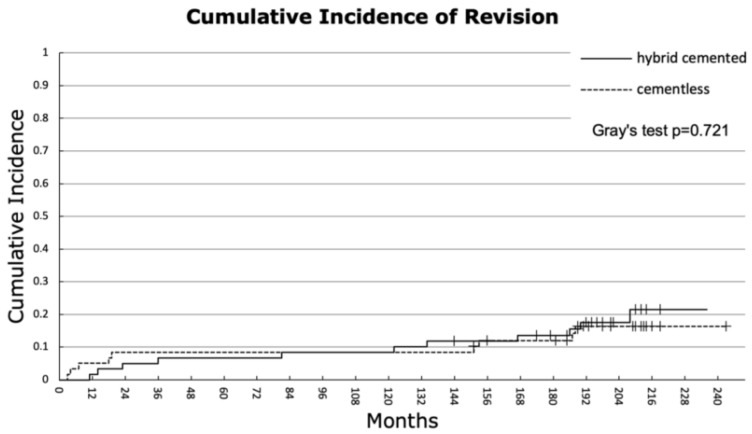
Overall cumulative incidence of revision. The hybrid cemented group showed an incidence of 6.7, 8.4, 13.5, and 21.5% after 5, 10, 15 years, and at the end of follow-up (19.6 years). For the cementless group, the cumulative incidences were 8.4, 8.4, 12.0, and 16.3% after 5, 10, 15 years, and at the end of the follow-up (20 years) (*p* = 0.721).

**Figure 3:**
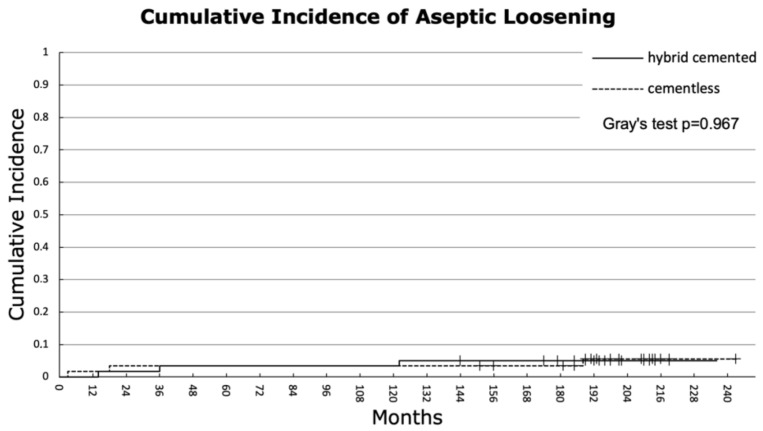
Cumulative incidence of aseptic loosening. The hybrid cemented group showed an incidence of 3.4, 3.4, 5.1, and 5.1% after 5, 10, 15 years, and at the end of follow-up (19.6 years). For the cementless group, the cumulative incidences were 3.4, 3.4, 3.4, and 5.5% after 5, 10, 15 years, and at the end of the follow-up (20 years) (*p* = 0.967).

Competing risk analysis revealed that the overall 10-year cumulative incidence of revision was 8.4% for both groups, increasing to 13.5% for the hybrid cemented group and 12.0% for the cementless group at 15 years. At the final follow-up, the cumulative incidences were 21.5% for the hybrid cemented group (at 19.6 years) and 16.3% for the cementless group (at 20 years). Gray’s test confirmed no significant difference in overall cumulative revision incidence between the two fixation methods (*p* = 0.721) (Figure 2). For the endpoint aseptic loosening, both groups demonstrated an identical 10-year cumulative incidence of 3.4%. At final follow-up, the hybrid cemented group exhibited a cumulative incidence of 5.1% (at 19.6 years), compared to 5.5% for the cementless group (at 20 years), with no significant difference observed between the groups (*p* = 0.967) (Figure 3).

### 3.4. Clinical Outcomes

At a minimum of 10 years postoperatively, clinical and functional outcomes were evaluated in 42 patients (46 TKAs), after excluding 13 TKAs (10 revisions and 3 for health reasons). Of the evaluated cases, 22 TKAs (47.8%) were hybrid cemented and 24 TKAs (52.2%) were cementless implants. Specifically, the cementless group’s mean total KSS increased from 96.52 preoperatively to 168.63 at 10 years, while the hybrid cemented group improved from 91.52 to 168.18. Although a significant difference was noted in the preoperative clinical KSS (54.10 for cementless vs. 45.40 for hybrid cemented; *p* = 0.011), no significant differences were observed between the groups in clinical, functional, or total KSSs at the 10-year follow-up (*p* = 0.736, *p* = 0.948, and *p* = 0.480, respectively) (Table 4).

### 3.5. Radiological Outcomes

**Figure 4:**
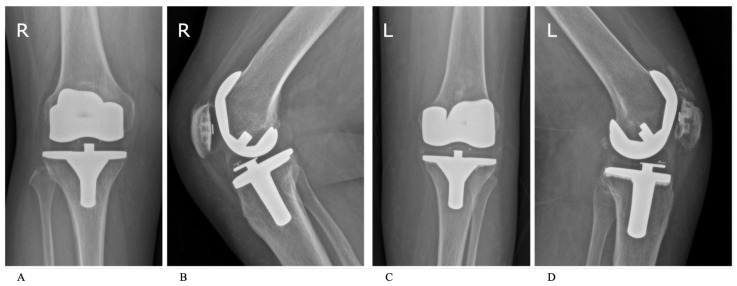
Anteroposterior and lateral radiographs of cementless (**A**,**B**) and hybrid cemented (**C**,**D**) TKA implants, 10 years postoperatively. “R” indicates the right knee; “L” indicates the left knee.

In the radiological evaluation, no evidence of osteolysis was detected in either group. Radiolucent lines between 1 and 2 mm were found in 3 TKAs of 2 patients in the cementless group, but none in the hybrid cemented group. However, there was no significant difference (*p* = 0.075) in the incidence of loosening between the two techniques at a minimum follow-up of 10 years (Figure 4).

## 4. Discussion

The optimal fixation method in TKA—cementless versus cemented—remains a subject of ongoing debate. With the increasing adoption of cementless TKAs, it is essential to comprehensively evaluate their long-term performance to inform future surgical decision-making [16]. Historically, cemented TKAs have demonstrated superior implant survivorship compared to cementless alternatives; however, recent advancements in implant design have aimed to close this gap [5].

The main finding of this study was that cementless and hybrid cemented TKAs with the same implant design exhibited comparable long-term clinical, radiographic, and revision outcomes at a minimum follow-up of 10 years. The nearly identical cumulative incidence rates of revision and aseptic loosening observed between the two fixation methods suggest that modern design improvements, such as bioactive coatings and enhanced geometries, have effectively addressed the historical concerns regarding cementless fixation. These results align with recent literature documenting similar survival and functional outcomes between cementless and cemented (or hybrid) TKA systems [17]. For instance, Zhou et al. [18] reported no significant difference in survival in a meta-analysis of 812 TKAs, while Kim et al. [19] observed similar survivorship in bilateral TKAs with a mean follow-up of 16.6 years. Furthermore, research by Kim et al. [20] demonstrated survival rates of 97% for cementless and 98% for cemented implants over 23.8 years. More recently, Gibon et al. [21] found that both cementless and hybrid monoblock tibial components achieved 96% implant survivorship at 10 years, exceeding the 89% survivorship observed in the traditional cemented tibial components.

However, an increased revision rate was observed in both groups toward the end of the follow-up period. This increase is likely attributable to the “floating platform” design, which has previously been associated with a higher incidence of inlay fractures and represented the primary cause of prosthesis failure in our cohort [22]. While short- to mid-term results of this implant have generally demonstrated encouraging outcomes, recent literature has highlighted concerns regarding long-term durability, specifically noting an increased revision rate linked to inlay fractures under mechanical stress conditions such as deep knee flexion combined with torsional loading [22,23,24]. For example, Yoon et al. [22] reported insert fractures as a distinct failure mechanism in the e.motion floating-platform design, recommending patient counseling about activity restrictions to minimize this risk. In this context, our study provides important long-term follow-up data, reinforcing the durability concerns raised in the literature.

Another noteworthy finding was the significantly shorter operative time in the cementless group (*p* = 0.017), which may reduce intraoperative complications and potentially lower the risk of periprosthetic joint infections. This trend is further supported by the absence of infection-related revisions in the cementless group, although the difference did not reach statistical significance (*p* = 0.087).

Furthermore, both cementless and hybrid cemented TKAs achieved substantial improvement in clinical outcomes, as reflected by the significant increase in KSSs in both groups from baseline. Importantly, no significant differences in clinical scores were observed between the two groups at the 10-year follow-up (*p* = 0.480), indicating that cementless fixation does not compromise functional outcomes. These findings are consistent with previous research, including a systematic review and meta-analysis by Anoop K et al. [25], which reported no significant differences in functional scores between cemented and cementless TKAs (*p* > 0.05)

Radiological outcomes further support the equivalence of these fixation methods. Radiolucent lines were observed in three tibial components in the cementless group, whereas none were found in the hybrid cemented group. These radiolucencies were non-progressive, did not correlate with implant failure or clinical symptoms, and the difference between groups was not statistically significant (*p* = 0.075). As reported by Costales et al. [26], radiolucent lines are a common occurrence after cementless TKA.

### Limitations

This study has several limitations. It was retrospective, which may have introduced sample bias due to loss to follow-up. The relatively modest sample size may limit the statistical power to detect differences in rare outcomes. The study compared cementless TKAs with hybrid cemented TKAs. However, recent studies indicate that there is no significant difference in survival, clinical scores, or radiological outcomes between cemented and cementless femoral components [27]. Furthermore, the study used TKAs with a unique rotating platform known as the “floating platform”, which was associated with a higher rate of inlay fractures in later studies and was subsequently withdrawn by the manufacturer due to this issue [22]. However, the same TKAs were used in both groups, resulting in PE wear in both groups.

## 5. Conclusions

In conclusion, this study demonstrated that cementless and hybrid cemented fixation techniques for TKA, using the e.motion posterior cruciate-retaining floating-platform design, achieved comparable clinical, radiological, and survival outcomes at a minimum 10-year follow-up. Notably, revision rates increased toward the end of follow-up in both groups, primarily due to polyethylene insert failures associated with the floating-platform design, highlighting existing concerns regarding its long-term durability. Aseptic loosening rates, the primary endpoint, however, remained low and comparable across the two fixation methods. Future research should investigate specific patient demographics, variations in implant designs, and evolving surgical techniques to further refine and optimize fixation strategies in TKA.

## Figures and Tables

**Table 1:** Preoperative demographic data of 120 included TKAs, 60 TKAs of the cementless group, and 60 TKAs of the hybrid cemented group.

Demographic Data	All Patients *n* = 120	Cementless *n* = 60	Hybrid Cemented *n* = 60	*p*-Value
Mean patient age, years (range)	66.93 (33 to 90)	65.72 (43 to 85)	68.13 (35 to 90)	0.189 *
Female patients, *n* (%)	81 (67.50)	35 (58.33)	46 (76.67)	0.032 ^†§^
Mean BMI kg/m^2^ (range)	30.28 (19 to 51)	30.03 (19 to 44)	30.53 (23 to 51)	0.615 *
L–R knees	56:64	29:31	27:33	0.714 ^†^
Operative time, minutes (range)	130.71 (80 to 200)	125.7 (80 to 175)	135.7 (95 to 200)	0.017 *^§^
Primary diagnosis, *n* (%)				
Idiopathic arthritis	106 (88.3)	52 (86.7)	54 (90.0)	0.570 ^†^
Posttraumatic arthritis	6 (5)	5 (8.3)	1 (1.7)	0.094 ^†^
Rheumatoid arthritis	4 (3.3)	2 (3.3)	2 (3.3)	1.000 ^†^
Avascular necrosis	1 (0.8)	1 (1.7)	0 (0)	0.331 ^†^
Unicondylar knee arthroplasty	3 (2.5)	0 (0)	3 (5)	0.079 ^†^

* Student’s *t*-test; † Chi-square test; § Statistically significant; BMI, Body Mass Index; L, left; R, right.

**Table 2:** Demographic data of the evaluable cases (*n* = 59) by fixation method (cementless vs. hybrid cemented).

Demographic Data	All Patients *n* = 59	Cementless *n* = 29	Hybrid Cemented *n* = 30	*p*-Value
Mean patient age, years (range)	62.8 (35 to 78)	61.8 (49 to 77.5)	63.8 (35 to 78)	0.234 ^a^
Female patients, *n* (%)	46 (78%)	23 (79.41%)	23 (76.7%)	0.807 ^†^
Mean BMI kg/m^2^ (range)	30.3 (19 to 46)	30 (19 to 37)	30.5 (23 to 46)	0.915 ^a^
L–R knees	26:33	12:17	14:16	0.683 ^†^
Operative time, minutes (range)	130.3 (80 to 190)	125.4 (80 to 175)	135.0 (98 to 190)	0.162 ^a^
Primary diagnosis, *n* (%)				
Idiopathic arthritis	54 (91.6)	28 (96.5)	26 (86.7)	0.353 ^†^
Posttraumatic arthritis	1 (1.7)	0 (0)	1 (3.3)	1.000 ^†^
Rheumatoid arthritis	3 (5)	1 (3.5)	2 (6.7)	1.000 ^†^
Avascular necrosis	0 (0)	0 (0)	0 (0)	-
Unicondylar knee arthroplasty	1 (1.7)	0 (0)	1 (3.3)	1.000 ^†^

^a^ Mann–Whitney Test; † Chi-square test; BMI, Body Mass Index; L, left; R, right.

**Table 3:** Causes of revision and median time to revision with a minimum 10-year follow-up.

Reasons for Revision	All Patients (*n* = 120)	Cementless (*n* = 60)	Hybrid Cemented (*n* = 60)	*p*-Value
Polyethylene failure, *n* (%)	7 (35)	3 (15)	4 (20)	0.761 ^a^
Aseptic loosening, *n* (%)	6 (30)	3 (15)	3 (15)	0.967 ^a^
PJI, *n* (%)	3 (15)	0 (0)	3 (15)	0.087 ^a^
Limited mobility, *n* (%)	4 (15)	3 (15)	1 (5)	0.299 ^a^
Total, *n* (%)	20 (100)	9 (45)	11 (55)	0.721 ^a^
Time to Revision				
Polyethylene failure, months (median, IQR)	176.5 (151.5 to 187.7)	152 (151.0 to 161.5)	186 (167.0 to 190.0)	0.761 ^a^
Aseptic loosening, months (median, IQR)	18.0 (8.5 to 79.0)	10.5 (3 to 60.5)	25 (14.0 to 57.5)	0.967 ^a^
PJI, months (median, IQR)	46.0 (11 to 94.2)	0 (0 to 0)	46 (11.0 to 94.2)	0.087 ^a^
Limited mobility, months (median, IQR)	7.0 (4.0 to 19.0)	5.5 (4.0 to 10.0)	23 (23.0 to 23.0)	0.299 ^a^
Overall time to revision (median, IQR)	81.0 (14.0 to 167.0)	18.5 (4.7 to 152.5)	101.5 (20.7 to 171.7)	0.721 ^a^

^a^ Gray’s test.

**Table 4:** Clinical outcomes by follow-up interval.

KSS Outcome (Range)	Cementless (*n* = 24)	Hybrid Cemented (*n* = 22)	*p*-Value
Mean clinical score			
Preoperative	54.10 (10 to 95)	45.40 (0 to 100)	0.011 *§
5-year follow-up	93.05 (55 to 100)	89.17 (45 to 100)	0.109 *
10-year follow-up	89.25 (65 to 100)	88.18 (58 to 99)	0.736 *
Mean functional score			
Preoperative	42.45 (0 to 69)	46.10 (10 to 77)	0.180 *
5-year follow-up	91.69 (64 to 100)	90.52 (63 to 100)	0.492 *
10-year follow-up	80.42 (30 to 100)	80.00 (30 to 100)	0.948 *
Mean total score			
Preoperative	96.52 (0 to 153)	91.52 (44 to 142)	0.291 *
5-year follow-up	184.75 (144 to 200)	179.68 (113 to 200)	0.146 *
10-year follow-up	168.63 (104 to 197)	168.18 (93 to 199)	0.480 *

* Student’s *t*-test; § Statistically significant; KSS, Knee Society Score.

## Data Availability

The original contributions presented in this study are included in the article. Further inquiries can be directed to the corresponding author(s).

## Abbreviations

The following abbreviations are used in this manuscript:

TKA	Total knee arthroplasty
KSS	Knee Society Score
BMI	Body Mass Index
IQR	Interquartile range
PE	Polyethylene
PJI	Periprosthetic joint infection
RCT	Randomized controlled trial
SD	Standard deviation
